# Community-acquired meningitis caused by a CG86 hypervirulent *Klebsiella pneumoniae* strain: first case report in the Caribbean

**DOI:** 10.1186/s12879-016-2065-2

**Published:** 2016-12-07

**Authors:** Bénédicte Melot, Sylvain Brisse, Sébastien Breurec, Virginie Passet, Edith Malpote, Isabelle Lamaury, Guillaume Thiery, Bruno Hoen

**Affiliations:** 1 Inserm-CIC 1424 et Service de Maladies Infectieuses et Tropicales, Centre Hospitalier Universitaire de Pointe-à-Pitre, Pointe-à-Pitre, France; 2 Microbial Evolutionary Genomics, Institut Pasteur, 28 rue du Dr Roux, 75724 Paris, France; 3 UMR3525, CNRS, Paris, France; 4 Laboratoire de Microbiologie clinique et environnementale, Centre Hospitalier Universitaire de Pointe-à-Pitre/les Abymes, Pointe-à-Pitre, France; 5 Unité Environnement et Santé, Institut Pasteur de Pointe-à-Pitre, Pointe-à-Pitre, Guadeloupe France; 6 Faculté de Médecine Hyacinthe Bastaraud, Université des Antilles et de la Guyane, Pointe-à-Pitre, Guadeloupe France; 7 Service de Réanimation, Centre Hospitalier Universitaire de Pointe-à-Pitre/les Abymes, Pointe-à-Pitre, France; 8 Centre Hospitalier Universitaire de Pointe-à-Pitre, Service de Maladies Infectieuses et Tropicales, BP 465, 97159 Pointe-à-Pitre Cedex, France

**Keywords:** Community acquired meningitis, ST86 hypervirulent *Klebsiella pneumoniae* strain, Caribbean

## Abstract

**Background:**

Community-acquired bacterial meningitis due to *Klebsiella pneumoniae* has mainly been described in Southeast Asia and has a poor prognosis. Severe invasive infections caused by *K. pneumoniae*, including meningitis, are often due to hypervirulent strains *(hvKP),* which are characterized by capsular serotypes K1 and K2, a gene responsible for hypermucoviscosity, and the cluster for synthesis of the siderophore aerobactin.

**Case presentation:**

A 55 year old man with a history of essential hypertension, benign prostate hyperplasia, hyperlipidemia, obstructive sleep apnea, and chronic alcoholism was admitted for meningitis due to *Klebsiella pneumoniae* with a wild-type susceptibility profile. Its genomic features were consistent with a capsular K2 strain belonging to clonal group 86 (CG86) displaying the large virulence of *Klebsiella* plasmid (pLVPK) with heavy metal resistance gene clusters, aerobactin, *rmpA.*

**Conclusion:**

This is the first case of community-acquired meningitis caused by a hypervirulent strain of *hvKP* ever reported in the Caribbean.

## Background

*Klebsiella pneumoniae* is an uncommon cause of community-acquired bacterial meningitis [1, 2]. A new group of severe infections due to hypervirulent strains of *Klebsiella pneumoniae* has been described in the past few years, mainly in Southeast Asia, including liver abscesses, pneumonia, meningitis, metastatic localisations [2, 3] and has a poor prognosis [4]. Severe invasive infections caused by *K. pneumoniae*, including meningitis, are often due to *hvKP.* The genomic analysis of those strains most frequently highlights capsular serotypes K1 and K2 and the presence of a large virulence plasmid harboring the regulator of mucoid phenotype gene *rmpA*, responsible for hypermucoviscosity, and the cluster for synthesis of the siderophore aerobactin [5–7].

A 55-year-old man was admitted to the emergency room of the University Hospital of Pointe-à-Pitre, Guadeloupe, French West Indies, on July 14th, 2014, because of consciousness deterioration and photophobia. He was born and had always lived in Guadeloupe. He had a history of essential hypertension, benign prostate hyperplasia, hyperlipidemia, obstructive sleep apnea, and chronic alcoholism but he neither had cirrhosis nor diabetes. On July 10th, he was given amoxicillin-clavulanate orally for an acute suppurative left otitis media without microbiological documentation. At the time of admission, he was afebrile and hemodynamically stable, but otoscopy revealed a persisting suppurative otitis media and neurological examination showed a stiff neck. White blood cell count was 11.6G/L and serum C-reactive protein was 414 mg/L. Computed tomography (CT) scan of the brain was normal. Cerebrospinal fluid (CSF) was cloudy, with the following abnormalities: glucose below limit of detection, protein 8.38 g/L, and 5,500 white blood cells (WBC)/mm^3^, (90% of polymorphonuclear (PMN) cells). Many Gram-negative bacilli were observed on direct microscopy and CSF culture grew hypermucoviscous KP with a wild-type susceptibility profile. Immediately after lumbar puncture, intravenous cefotaxime (4 g qid) was started and the patient was transferred to the intensive care unit. CT-scan of the brain showed a left mastoiditis and CT-scan of the abdomen was normal, with no liver abscess. On July 16th, brain magnetic resonance imaging (MRI) FLAIR sequences showed diffuse subcortical hypointense signal abnormalities of the supratentorial zone, which was consistent with a posterior reversible encephalopathy syndrome without edema probably related to undertreated high blood pressure and exacerbated by the sepsis. On July 18th, repeat lumbar puncture yielded cloudy CSF with improved parameters: glucose 4.8 mmol/L, protein 3.0 g/L, 3,093 WBC/mm^3^ (87% of PMN cells). A few Gram-negative bacilli were noted on direct microscopy but the culture remained negative. Cefotaxime (4 g qid) was continued for a total of 21 days and the patient recovered progressively. When he was discharged from hospital, he still had multiple paresis of cranial nerves (III, VI, and VII) and hemiparesia. When he was re-evaluated 3 weeks later all symptoms had disappeared and neurological examination had returned to normal.

## Case presentation

To determine the genotypic characteristics of the KP isolate (SB4936), a genomic sequence was obtained using an Illumina 2 x 300 nt paired end protocol on a MiSeq instrument. Reads were assembled de-novo using CLCbio assembler. The genomic sequence was submitted to the European Nucleotide Archive (accession number PRJEB9692). Contigs were scanned using the BIGSdb tool (http://bigsdb.pasteur.fr) for core genome multilocus sequence typing (cgMLST) as well as KP virulence and resistance genes [8, 9]. Phylogenetic analysis of gene sequences showed that the strain belongs to KP sensu stricto [10]. Consistent with this, the genome sequence harbored the marker Kp50233, previously shown to be specific for KP [11]. Analysis of the 7-gene Multilocus sequence typing (MLST) sequences [12] showed that the isolate belongs to sequence type (ST) 86, the archetype ST of clonal group (CG) 86, previously recovered from severe community-acquired infections [9, 13]. Furthermore, comparisons of the 694 cgMLST genes revealed only 19 allelic mismatches when compared to reference strain SA1 of ST86, which demonstrates that isolate SB4936 belongs to CG86 [9, 13]. Consistently, the genome also harbored genes *kpiA* and *nikA2*, which are typical for this clonal group [11]. It also possessed the following virulence factors: *rmpA* and *rmpA2* (regulator of mucoid phenotype, associated with the hypermucoviscous phenotype), *iroBCDN* (coding for salmochelin), *iucABCDiutA* (coding for the aerobactin siderophore cluster), *kvg*AS (a two-component system), *mrkABCDFHIJ* (coding for the cluster for type III fimbriae involved in adhesion and biofilm formation). Conversely the *kfuABC* (iron aquisition) and *allABCDRS* (allantoin utilization) clusters were absent from its genome. The strain also possessed gene sequences *wzi-2* and *wzc-2*, previously shown to be specifically encountered in strain of capsular serotype K2, another important virulence factor of KP. The strain did not harbor the *irp1* and *irp2* genes coding for polyketide synthase/non ribosomal peptide synthetase associated with yersinibactin siderophore synthesis. No resistance gene other than *bla*_SHV-1_ was detected in the genome of SB4936 which was consistent with the antimicrobial susceptibility profile (resistance to only ampicillin, ticarcillin and piperacillin). The genomic features of isolate SB4936 were consistent with a hypervirulent strain with a wild-type susceptibility profile, as previously described for isolates of clonal group CG86 [8, 9, 11, 14]. The strain also harbored the heavy metal resistance gene clusters *pbr* (lead), *pco* (copper), *sil* (silver) and *ter* (tellurium). The presence of these clusters as well as of *rmpA* and the aerobactin cluster demonstrates the presence in SB4936 of a plasmid similar to pLVPK, the large virulence plasmid of *K. pneumoniae* [5]. Overall, 249 out of 251 protein-coding genes of pLVPK were present in SB4936, among which 237 were 100% identical in nucleotide sequence.

## Conclusion

We report the second case of invasive infection due to a hypervirulent community-acquired strain of KP ever identified in the Caribbean area [15] and the first case of community-acquired meningitis, possibly signaling its emergence in this part of the world.

The clinical presentation was similar to that of other published cases of meningitis caused by this strain [3, 7, 16]. Diabetes mellitus, liver cirrhosis and alcoholism have all been reported as the most significant risk factors for *K. pneumoniae* invasive severe infections [1, 2]. As in most other cases of meningitis due to hypervirulent KP it was community-acquired [7, 16]. This case report demonstrates that hypermucoviscous strains of *K. pneumoniae* have spread to the Caribbean. Therefore we would recommend that clinicians as well as microbiologists from this region be prepared to specifically investigate strains isolated from patients presenting with severe invasive community-acquired *K. pneumoniae* infections.

## Abbreviations

CG: Clonal group
cgMLST: Core genome multilocus sequence typing
CSF: Cerebro-spinal fluid
CT: Computed tomography
HvKP: Hypervirulent Klebsiella pneumoniae
KP: Klebsiella pneumoniae
MRI: Magnetic resonance imaging
pLVPK: Large virulence plasmid of Klebsiella Pneumoniae
PMN: Polymorphonuclear leucocytes
ST: Sequence type
WBC: White blood cells
